# E-waste recycling in an optimized way for copper recovery by leaching and a case study on E-waste generation and management in Dhaka city

**DOI:** 10.1016/j.heliyon.2024.e41453

**Published:** 2024-12-27

**Authors:** Kaniz Fatema, Md Niamul Hassan, Sanjida Hasan, Hridoy Roy

**Affiliations:** Department of Chemical Engineering, Bangladesh University of Engineering and Technology (BUET), Dhaka, 1000, Bangladesh

**Keywords:** E-Waste generation, PCB recycling, Leaching, Copper recovery, E-Waste management

## Abstract

The widespread adoption of electronic devices has enhanced living standards but has also led to a surge in electronic waste (e-waste), creating serious environmental and health challenges. Although various methods exist to recover valuable metals from e-waste, each has notable drawbacks. Among these, chemical leaching with aqua regia is widely used but is both highly corrosive and hazardous. This study introduces a safer, more environmentally friendly approach to copper recovery from e-waste using an iron-based leaching solution. A combination of experimental procedures and computational modeling was employed to optimize copper extraction from printed circuit boards (PCBs). The experiments involved treating PCBs with iron-based solutions of different concentrations and testing the effectiveness over two distinct time periods. The most effective recovery rate, 72.69 % over five days, was achieved using a 50:50 mixture of ferrous and ferric sulfate. Computational analysis with Python's SciPy library further identified 5.92 g of PCB as the ideal input quantity for the process. In addition to the lab-based work, a survey of Dhaka's primary e-waste recycling hubs, Nimtoli and Elephant Road, revealed that approximately 1173 tons of e-waste are processed in these areas each year. Based on experimental findings, the survey findings have a projection to generate over 35 million BDT annually through copper recovery. However, despite government initiatives to regulate e-waste management, unsafe handling practices remain widespread. These practices not only endanger workers and the environment but also hinder regulatory efforts. The study emphasizes the urgent need for stricter regulations, greater public awareness, and the adoption of eco-friendly methods, like the proposed iron-based solution, to ensure safer and more effective copper recovery.

## 1 Introduction

The rapid rise in electronic devices like TVs, computers, mobile phones, and washing machines has significantly improved living standards through technological innovation. However, this growth has also raised concerns about electronic waste (e-waste) and its byproducts. This research focuses on reviewing e-waste recycling methods, with an emphasis on optimizing copper recovery from printed circuit boards (PCBs) using less corrosive chemical leaching agents. Laboratory experiments inform the development of a Python-based model, utilizing the SciPy library, to maximize metal recovery. Additionally, field surveys and economic analyses examine the current state of e-waste collection and management in Dhaka, identifying areas for improvement and recommending strategies to address the global e-waste challenge effectively.

**Fig. 1:**
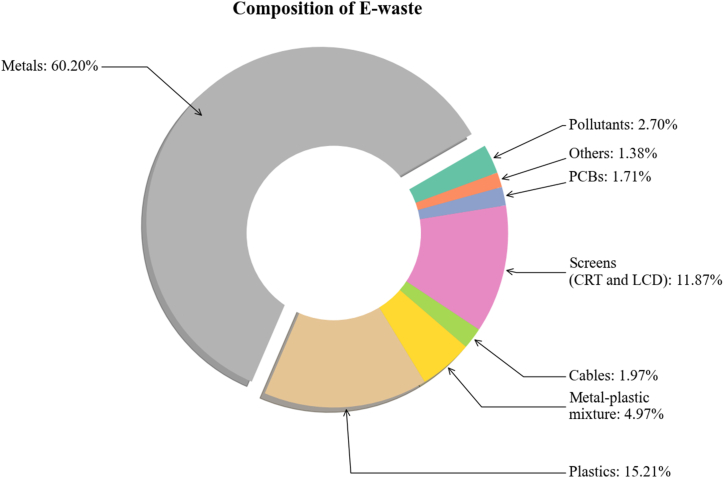
Composition of e-waste components [4].

E-waste, which consists of discarded electronic devices such as phones, TVs, and appliances, is increasing due to rapid technological progress and consumers' preference for the latest gadgets. From 2014 to 2019, global e-waste surged by 21 %, reaching 53.6 Mt, with projections estimating a further increase to 74 Mt by 2030 [1]. According to Kumar's review, around 70 % of the e-waste stems from private and public sectors [2]. Although big institutions and government organizations, to some extent, are bound to dispose of their waste to the proper dealer, household waste is freely disposed of by local ragpickers where it is handled improperly, and it leads to several adverse health effects on the recyclers [3]. E-waste contains a range of materials, such as plastics, metals, and glass, some of which can be systematically recovered, thus making this waste stream a resource of raw materials [4]. The overall composition is shown in Fig. 1. Fortunately, the metal part is 60 %, a considerable amount to be extracted and reused. Fig. 1 shows the composition of different elements in e-waste.

**Fig. 2:**
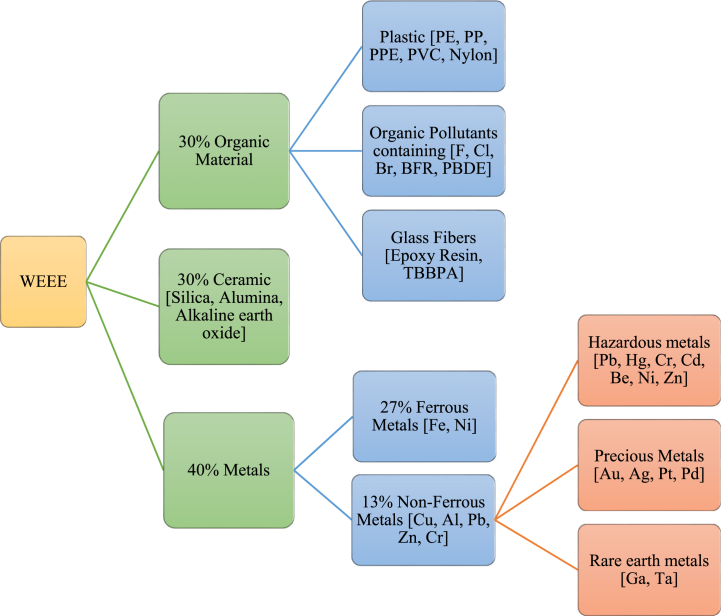
Constituents of waste electric and electronic equipment (WEEE) [8].

E-waste contains a mix of hazardous, non-hazardous, and valuable metals. Non-hazardous metals commonly found include copper (Cu), aluminum (Al), tin (Sn), manganese (Mn), and iron (Fe). Whereas hazardous substances such as sulfur (S), cadmium (Cd), beryllium oxide (BeO), brominated flame retardants, lead (Pb), lithium (Li), nickel (Ni), and mercury (Hg); chlorofluorocarbon; organic substances; infrequent earth elements such as yttrium (Y), lanthanum (La), cerium (Ce), neodymium (Nd), gallium arsenide (GaAs), and zinc sulfide (ZnS); halogenated compounds; and radioactive substances such as americium; and toner dust can also be present in e-waste [5,6]. In addition, plastic, glass, ceramics, wood etc. contribute to the non-metallic part of e-waste [7]. Fig. 2 categorizes the metal content along with the organic and ceramic elements of waste electric and electronic equipment (WEEE). Metals like iron and nickel make up over a quarter of the metallic fraction, with the rest consisting of plastics, other metals, glass, and ceramic materials. Fig. 2 illustrates the categorization of components in electronic wastes along with their composition.

The global demand for precious metals Pt, Au, Ag, and Pd, along with the base metals Zn, Pb, Cu, and Ni, and rare earth minerals Yt, La, Ce, and Nb, has recently increased significantly in electronic applications [9]. E-waste typically contains over 60 different metals, spanning valuable, precious, base, and rare earth metals, and is considered valuable due to its total metal content [10]. It was reported in London that the total value share was ranging from 85 % (PCBs) to 93 % (mobile phones) using precious metals (Pt, Pd, Au, Ag), while contributing less than 10 % of Fe, Al, Cu, and other base metals [11]. According to Borthakur and Govind, 2017, the potential revenue of PCBs was $21,200/ton with significant metal concentrations [12]. In 2020, an estimated amount of 12.5 MT Cu could potentially be recovered, which had a market value of US$ 6.45 per kg of Cu [11]. Therefore, a field survey is necessary to get valuable insights into e-waste accumulation and its potential to generate revenue by recovering metals using proven lab methods.

E-waste, which contains both metallic and non-metallic elements, including hazardous and valuable metals, poses serious environmental and health risks if not properly managed [[13], [14], [15]]. When e-waste is recycled using unsound, informal activities, it can produce many hazardous toxicants that may pollute the air, soil, water, and dust. Informal e-waste recycling, prevalent in developing countries, often involves unsafe practices like open-air burning and acid baths to extract valuable metals. These methods release toxic chemicals into the environment, leading to contamination of local ecosystems and posing health risks to nearby communities. For instance, in areas like Agbogbloshie, Ghana, and Guiyu, China, such practices have resulted in elevated levels of lead and other toxins in the soil and water, adversely affecting human health.

The study by Ibrahim Issah and his team highlighted significant health impacts from e-waste recycling, including musculoskeletal issues, physical injuries, respiratory and cardiovascular symptoms, stress, hearing loss, hepatic damage, DNA damage, and epigenetic changes [16]. Main exposures were linked to dismantling and burning activities, metal biomarkers in blood, and particulate matter in breathing zones. Another study by Hongfei Hu et al. examines the levels of heavy metal(loid) pollution in the Lianjiang River over a decade and assesses the associated health risks to children in an e-waste recycling area. Their assessment reveals that while individual non-carcinogenic risks from heavy metal(loids) in water for children are generally below harmful levels, As and Sr pose notable health concerns, including developmental effects and reproductive risks [17]. Carcinogenic risks, however, are significant, with As, Cr, Ni, Pb, and Cd exceeding acceptable thresholds, particularly through dermal absorption, which contributes the most to cancer risk.

Along with the toxic heavy metals, e-waste contributes to organic pollutants, which are extremely harmful for human health. Primitive processing methods like manual dismantling, open burning, and plastic recycling significantly expose humans to organic pollutants such as flame retardants, PAHs (Polycyclic aromatic hydrocarbons), PCBs (Polychlorinated biphenyls), and dioxin-related compounds. PAHs are carcinogens [18] and cause oxidative damage to DNA and lipid content in people [19]. PCBs can accumulate in breast milk, exposing nursing infants to these harmful chemicals during a critical period of development. This exposure has been linked to potential adverse effects on infants, including impaired neurodevelopment, weakened immune function, and hormonal disruptions [20]. Short-term exposure to high levels of dioxins can cause skin lesions and altered liver function, while long-term exposure is linked to immune, nervous, endocrine, and reproductive system impairments. Classified as a known human carcinogen, dioxins pose the greatest risk to developing fetuses and newborns, with efforts needed to minimize exposure due to their high toxicity [21].

In addition to health risks, e-waste poses severe environmental threats. Owusu-Sekyere et al. investigated the environmental impacts of informal e-waste recycling in Agbogbloshie, Ghana, focusing on heavy metal and organic pollutant contamination in soil and groundwater [22]. The soil in e-waste recycling areas showed significant contamination with heavy metals like Cu, Cd, Sb, Pb, Zn, and Au, alongside organic pollutants such as PCBs, PBDEs, and PAHs, largely linked to dismantling and burning activities. High contamination degrees (Cdeg) and ecological risks (Ri) were observed, particularly at sites with elevated Sb, Cd, Pb, and oil-related pollutants. Groundwater samples indicated poor quality based on the CCME Water Quality Index and HPI, with heavy metal pollution in most sites where the highest organic pollution was 1.76 μg/L PAH 17 and 0.14 μg/L PCB. Besides water and soil, e-waste toxic dust particles were also found in the ambient air [20]. Table 1 lists the health risks and environmental impacts of e-waste constituents.

**Table 1:** Health risks and environmental hazards of e-waste components.

Pollutant	Source	Health impacts	Environmental impacts
**Heavy Metals (e.g., Pb, Cd, Cr, Ni, As)**	Battery, soldering, circuit boards	Neurological damage, developmental delays, kidney damage, carcinogenic risks through dermal absorption and ingestion.	Soil and water contamination, bioaccumulation in ecosystems.
**Polychlorinated Biphenyls (PCBs)**	Capacitors, transformers	Neurodevelopmental impairment, immune suppression, hormonal disruption, bioaccumulation in breast milk.	Persistent organic pollutant (POP), long-term contamination of soil and water.
**Flame Retardants (PBDEs)**	Plastic casings, wiring insulation	Hormonal disruption, neurotoxicity, potential reproductive issues.	Persistent in the environment, contamination of air and dust.
**Dioxins**	Burning plastics, PCB incineration	Skin lesions, liver dysfunction, carcinogenic, immune, nervous, and endocrine system damage.	Long-lasting environmental toxins, bioaccumulation in wildlife and food chains.
**Polycyclic Aromatic Hydrocarbons (PAHs)**	Open burning of e-waste	Carcinogenic, oxidative DNA damage, respiratory and cardiovascular health risks.	Air, soil, and water pollution, degradation of ecosystems.
**Arsenic (As)**	Semiconductor devices	Developmental effects, reproductive risks, carcinogenic.	Groundwater contamination, toxic to aquatic life.
**Lead (Pb)**	Circuit boards, soldering materials	Neurological issues, cognitive impairment, particularly harmful to children.	Soil and water contamination, leaching into ecosystems.
**Cadmium (Cd)**	Rechargeable batteries, resistors	Kidney damage, bone demineralization, carcinogenic.	Contamination of water and soil, bioaccumulation in crops and aquatic organisms.
**Copper (Cu)**	Wiring and circuit boards	Gastrointestinal issues, liver and kidney damage at high exposures.	Soil and water contamination, toxicity to aquatic organisms in high concentrations.

Addressing these issues requires strengthening formal recycling processes, enforcing regulations to prevent illegal e-waste dumping, and promoting the design of electronics with longer lifespans and safer materials. International cooperation and adherence to agreements like the Basel Convention are crucial in mitigating the adverse effects of e-waste on health and the environment. While global e-waste generation is rising rapidly as an expansion of communication, the dumping sites of the world, like Bangladesh are experiencing alarming health and environmental impacts. Despite this, only a small portion is collected for recycling, with most being improperly discarded. According to the *E-Waste Monitor 2019*, just 17.4 % of global e-waste was formally documented and recycled in an environmentally responsible manner in recent years, while e-waste production continues to outpace recycling efforts by nearly five times [23,24]. Although regulations like Bangladesh's *Hazardous Waste Management Rules* exist, weak enforcement underscores the urgent need for improved e-waste recycling and management strategies.

Given the severe consequences of e-waste and the dangers of unsafe recycling practices, this study introduces an innovative approach to repurposing e-waste. It utilizes a safer, less harmful iron-based leaching solution to extract copper, offering a sustainable alternative to the widely used but highly toxic aqua regia. In addition, by integrating laboratory findings with field survey data, it proposes an eco-friendly and economically viable business model for managing one of the world's most hazardous waste streams. This groundbreaking work demonstrates how innovation and sustainability can intersect to transform a global challenge into an opportunity for positive change.

## 2 A review of E-waste source and existing recovery techniques

### 2.1 The wealth within printed circuit boards

Printed circuit boards (PCBs) are a crucial part of electronic devices, acting as a platform that connects and supports components like microchips, resistors, and capacitors. Made from epoxy resin or fiberglass coated with thin copper films, PCBs enable electrical signal transmission between components [25]. Although PCBs account for at least 3 % of total e-waste by weight [26], they contain valuable metals such as gold, silver, copper, and palladium, alongside hazardous substances like gallium arsenide, beryllium, and brominated flame retardants [[27], [28], [29]].

**Fig. 3:**
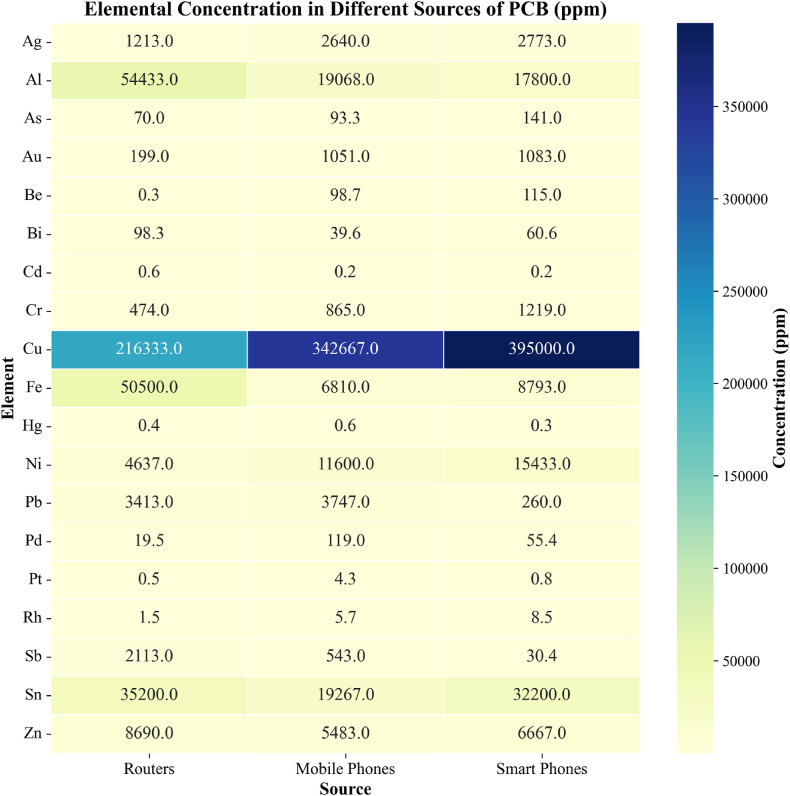
Elemental concentration of router, mobile phone, and smartphone [30].

A heat map in Fig. 3 highlights the elemental concentrations in PCBs from routers, mobile phones, and smartphones. Routers exhibit high levels of copper (216,333 ppm), aluminum (54,433 ppm), iron (50,500 ppm), and tin (35,200 ppm). Mobile phones show even greater copper concentrations (342,667 ppm) along with aluminum (19,068 ppm) and nickel (11,600 ppm). Smartphones have the highest copper content (395,000 ppm) and significant aluminum (17,800 ppm) and nickel (15,433 ppm) levels. The heat map visually emphasizes the abundance of metals like copper and aluminum across different devices.

**Fig. 4:**
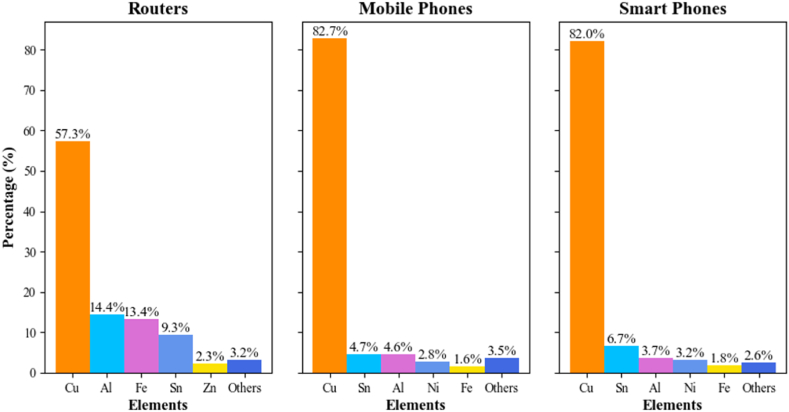
The top five elements in different sources of PCB.

Fig. 4 illustrates the maximum percentages of five elements present in three different PCB sources. routers, mobile phones, and smartphones. For routers, copper (Cu) constitutes 57.32 % of the total concentration, followed by aluminum (Al) at 14.42 %, iron (Fe) at 13.38 %, tin (Sn) at 9.33 %, and zinc (Zn) at 2.30 %. The remaining elements collectively account for 3.24 % of the total concentration. In mobile phones, copper is the most abundant element, making up 82.75 % of the total concentration, followed by tin at 4.65 %, aluminum at 4.60 %, nickel (Ni) at 2.80 %, and iron at 1.64 %. The remaining elements constitute 3.55 % of the total concentration. Similarly, in smartphones, copper also dominates with 82.01 % of the total concentration, followed by tin at 6.69 %, aluminum at 3.70 %, nickel at 3.20 %, and iron at 1.83 %. The remaining elements collectively account for 2.58 % of the total concentration. These percentages highlight the significant presence of copper across all three types of electronic devices, with aluminum, iron, tin, zinc, and nickel also being prominent, while the remaining elements contribute a smaller portion to the overall concentration.

### 2.2 Methods to leach out the metals from E-waste

The solid waste management techniques employed to address the growing issue of e-waste include landfilling, incineration, recycling, and reuse. Among these methods, recycling plays a particularly vital role, as it not only enables the recovery of valuable materials but also helps reduce the environmental impact associated with improper disposal. Different types of physical and chemical processes, such as mechanical processing, chemical leaching and bioleaching, pyrolysis, electrochemical methods, hydrometallurgical and bio-metallurgical processes are used to efficiently recover metals from e-wastes. These approaches come with several drawbacks as well.

Ankit and his team studied different processes of metal recovery with their advantages and disadvantages [3]. They found that electrochemical methods are environmentally friendly and energy-efficient, with minimal chemical use, while hydrometallurgical processes offer high efficiency and selectivity in metal recovery. Pyrometallurgy provides high recovery rates and low installation costs, and alkali smelting prevents toxic emissions, making these methods advantageous for metal extraction. These methods have drawbacks also which is presented in Table 2.

**Table 2:** Advantages and disadvantages of different e-waste recovery methods.

Process	Advantages	Disadvantages
**Electrochemical methods**	- Environmentally friendly - Energy-efficient - Minimal chemical use	- Multistep nature increases production costs
**Hydrometallurgical Processes**	- High efficiency in metal recovery - High selectivity	- Requires harsh chemicals - Generates hazardous waste - Demands extensive wastewater treatment - High costs - Complex processes that impact health, environment, and sustainability
**Pyrometallurgy**	- High recovery rates - Low installation costs - Minimal environmental impact	- High energy consumption - High installation costs - Oxidation issues
**Plasma Melting and Metal Trapping**	- Efficient processes	- High energy consumption - High installation costs
**Alkali Smelting**	- Prevents toxic emissions	- Challenges in metal separation due to low basicity
**Matte Collecting**	- Efficient in certain conditions	- Low efficiency in selective recovery

Leaching, particularly chemical and biochemical methods, offers advantages like lower energy consumption, selective metal recovery, and simpler equipment compared to processes like pyrometallurgy and smelting. It operates at ambient or mild conditions, reducing costs and environmental impact, and can be fine-tuned for specific metals, making it a sustainable alternative for e-waste management. Below are some examples of leaching procedures.

#### 2.2.1 Chemical leaching

Chemical leaching is favored for its high metal recovery rates and preservation of metal quality [31]. The choice of leaching agent, influenced by factors like metal properties, selectivity, and regeneration costs, is critical [32]. Common lixiviants like cyanide, thiourea, thiosulfate, and aqua regia yield varying metal recovery rates. Cyanide leaching, widely used for gold and silver extraction from PCBs, is effective but poses environmental and health risks [33]. Halide leaching, using chloride, bromide, or iodide, offers alternatives, with sodium chloride being suitable for gold and iodide providing a fast, non-toxic option [34,35].

Thiourea is historically used for extracting gold and silver. Jing-Ying showed that about 90 % of gold and 50 % of silver were leached by the reaction of 2 h [36]. Gold dissolution occurs through complex ion formation, with thiourea eventually oxidized into cyanamide and elemental sulfur. This method is effective but unstable in acidic environments [37]. Again, the thiosulfate process described by Murali, A. et al. utilizes an electrochemical reaction catalyzed by copper ions, where gold dissolves as aurous thiosulfate ($\text{Au}{{\left(S_{2}O_{3}\right)}_{2}}^{3-}$) on the anodic area [38]. Maintaining correct concentrations of ammonium and thiosulfate is crucial to prevent cupric ion reduction and ensure efficient gold dissolution. Thiosulfate leaching offers high selectivity, non-corrosiveness, and low toxicity but requires significant energy consumption during extraction [[39], [40], [41], [42]].

A very common and popular way to leach out metals is to use aqua regia. This process is capable of oxidizing metals and is commonly used in industrial metal determination processes. However, it is less favored due to its release of toxic gases such as nitrogen oxides and high reagent consumption [43]. Consequently, it poses health hazards, and it is now imperative to discover a novel extraction agent that addresses environmental and health concerns, ensuring an optimal extraction process. Zupanc and the team stated that the use of less corrosive alternatives such as iron solutions instead of traditional aqua regia mitigates environmental hazards and enables chemical leaching to emerge as a promising method [44].

#### 2.2.2 Bioleaching

**Fig. 5:**
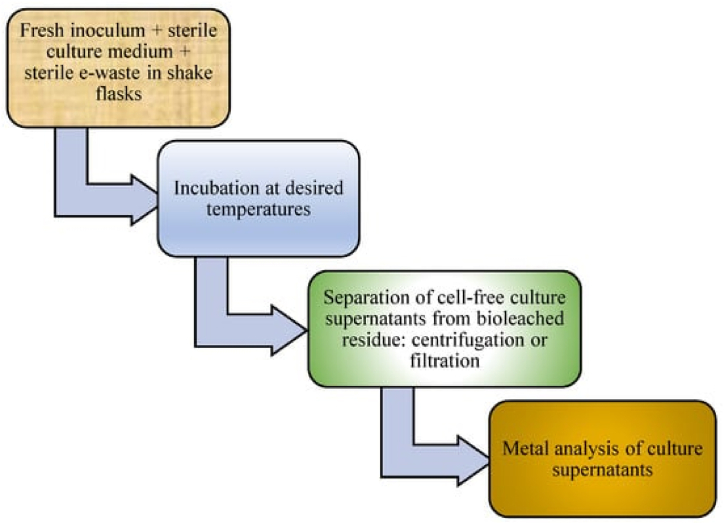
Schematic diagrams showing procedures for the one-step bioleaching of metals from e-waste [48].

Bioleaching, which uses microorganisms to extract metals, is valued for its sustainability and eco-friendliness, particularly in developed nations [45]. It employs microbes to oxidize and dissolve metals, converting metal sulfides into soluble sulfates. The process includes one-step, two-step, and spent-medium methods. In the one-step process, microbes grow directly in a mineral-containing medium with an inducer like glycine. The two-step method involves cultivating microbes to their logarithmic phase before adding minerals, while spent-medium bioleaching uses a cell-free cyanide solution derived from filtered bacterial cultures [46,47]. Fig. 5 shows the schematic for one-step bioleaching process.

The study by Arda Işıldar and the team provided a proof-of-concept of a two-step approach in metal bioleaching from PCB by bacterially produced lixiviants. They found that a two-step bioleaching of Cu and Au resulted in 98 % and 44 % efficiency, respectively [49]. Another study by Violaceum C. showed the high efficiency of gold bioleaching from treated electronic scrap materials containing 0.21 % Au and 3.67 % Cu [50]. Despite having the perks of eco-friendliness and sustainability, bioleaching is a bit challenging in regards of ensuring proper culturing of the microbe and management of the whole process.

Overall, both leaching methods contribute to metal recovery from e-waste, addressing the growing need for e-waste recycling in a sustainable way. While bioleaching is environmentally friendly and sustainable, it necessitates careful control of microbial growth and process conditions. In contrast, chemical leaching offers greater versatility and efficiency, despite potential environmental risks.

## 3 Methodology

PCBs contain around 26 % metal, including copper, lead, aluminum, and heavy metals like cadmium and nickel, with copper accounting for 10–30 % of their total mass, making it the most prevalent metal [51,52]. Copper, a key industrial metal due to its superior conductivity, is in high demand. This study hence focused on copper extraction from PCBs using aqua regia and an iron solution with ferrous and ferric ions. By optimizing the iron solution concentration and PCB-to-solution ratio, the authors developed a cost-efficient method to maximize copper recovery and minimize e-waste feed losses.

### 3.1 Collection of samples

Router PCB e-waste, purchased from a local e-scrap store in Chankharpool, Old Dhaka, at 50 Taka per PCB, was transported to the laboratory and manually stripped of non-metallic components. The cleaned PCBs were dried at 140 °C for 48 h, ground into a fine, homogeneous powder using an electronic blender, and stored in an airtight bag. Fig. S1 in the Supplementary data file shows the flow diagram of pretreatment of sample.

### 3.2 Experimental work

The study employed a systematic approach to optimize copper extraction from router PCBs using chemical leaching, with detailed procedures provided in the Supplementary data file. Table S1 and Table S2 presents the summary of material balance of relevant weights of the raw materials. Initially, PCB powder was dissolved in freshly prepared aqua regia, heated, and diluted to determine copper content using Atomic Absorption Spectrophotometry (AAS). This process is illustrated in Fig. S2. While aqua regia effectively extracted copper, its corrosive nature, toxic byproducts, and environmental risks highlighted the need for safer alternatives. To address this, a leaching agent composed of ferrous and ferric sulfate was developed, leveraging the reduced environmental impact of iron-based solutions. PCB powder was treated with this solution under controlled temperature and agitation, and copper recovery was monitored over a 10-day period, identifying 5 days as the optimal duration for extraction. Fig. S3 shows this process.

The composition of the iron solution was further refined by testing three different ratios of ferrous to ferric sulfate, with a 1:1 vol ratio achieving the highest copper recovery of 72.69 %. This step is presented in the flow diagram of Fig. S4. To maximize efficiency, the study also determined the optimal PCB-to-solution ratio, identifying 5.92 g of PCB per 500 mL of leaching agent as the ideal feed. Beyond this amount, the recovery rate diminished. Fig. S5 illustrates this method.

## 4 Results and discussion

### 4.1 Copper content of PCB and the optimum duration for leaching

The final solution containing PCB in aqua regia had a volume of 120 ml, where 80 ml of distilled water was added for dilution. The AAS showed that the concentration was 500 ppm for copper, which was 6 wt%. The value was taken as the standard of the copper content of PCB.

According to Stellan Holgersson et al., router PCBs contain 21.6 wt% copper [30]. The copper content in various fractions of computer and mobile phone PCBs ranges from 16 to 26.8 wt%, as reported in several studies [[53], [54], [55], [56]]. This range includes magnetic, conductive, mixed, and non-conductive parts of PCBs. In this study, the observed copper content was significantly lower than the values reported in the literature. This discrepancy is because the PCBs used in this study comprised only the metal parts, predominantly the conductive metals, and the samples were router PCBs rather than computer or smartphone PCBs.

For the recovery of copper from e-waste, a mixture of iron solutions was used. The leaching agent consisted of 200 mL of ferrous and ferric solutions having a 1:1 vol ratio with 0.5 molarity for both components. To find out the optimum duration for the extraction process, 10 g PCB was taken for leaching out copper, and the concentration was tested on the 5th day and the 10th day. The result showed that the amount of copper that could be leached out was 2.17 wt% on the 5th day when it increased slightly to 2.37 wt% on the 10th day. If the amount of recovered copper, 6 wt%, is considered as the copper content of the PCB, then the percent extraction of copper using the iron solution was 36.14 wt% on the 5th day and 39.45 wt% on the 10th day. Thus, the iron solution could extract more than one-third of the copper in the PCB sample, but given the insignificant improvement with doubling the leaching time, subsequent experiments were conducted over a 5-day timeframe.

### 4.2 Copper leaching - impact of iron solution composition

**Fig. 6:**
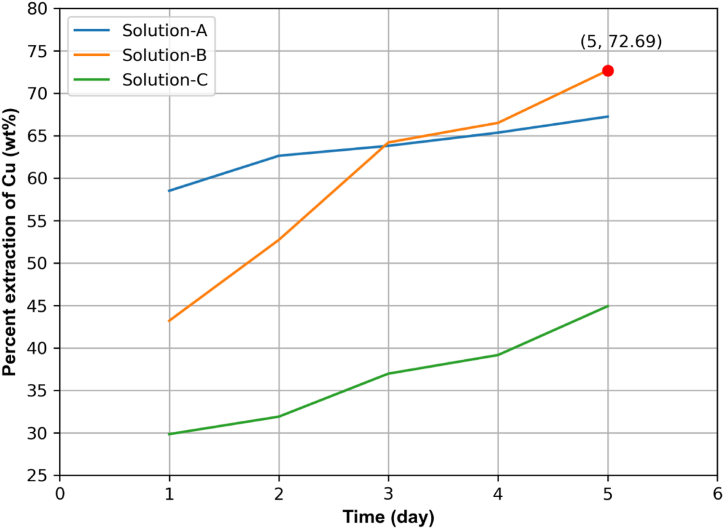
Concentration of extracted Cu vs. time for iron solution of different compositions.

Applying the optimum duration for leaching, this study optimized the volume ratio of iron solutions for which the maximum amount of copper could be extracted. The graph in Fig. 6 illustrates the percent extraction of copper (Cu) for 5 days using three different solutions (Solution A, Solution B, and Solution C) with varying volume ratios of ferrous sulfate (Fe(II)) and ferric sulfate (Fe(III)). The data for this process is presented in Table S3 in the Supplementary data file. The horizontal axis represents the duration of the leaching process in days, while the vertical axis represents the percentage of copper extracted from the solution. Solution A (25 % Fe(II) and 75 % Fe(III)) shows a steady increase in copper extraction from 58.52 % to 67.26 % over the 5 days. Solution B (50:50 ratio of Fe(II) to Fe(III)) starts at 43.22 % and rises significantly to 72.69 % by the fifth day. Solution C (75 % Fe(II) and 25 % Fe(III)) begins at about 29.85 % and steadily climbs to 44.93 % by the end of the period.

Among the three solutions, Solution B demonstrates the highest extraction efficiency, achieving 72.69 % copper extraction on the fifth day. This indicates that the equal parts combination of Fe(II) and Fe(III) is the most effective for copper extraction. Solution A, with a lower proportion of Fe(II), shows a slower increase in extraction efficiency, suggesting that this ratio is less optimal. Solution C, which has the highest proportion of Fe(II), achieves the lowest extraction percentage, indicating that a higher proportion of Fe(II) might not be as efficient for copper extraction.

The data indicates that the extraction of copper improves with prolonged exposure to the iron solutions across all tested ratios. However, the effectiveness of the extraction process is significantly influenced by the specific ratio of ferrous to ferric sulfate. Therefore, Solution B is identified as the optimal solution for maximizing copper extraction efficiency, providing valuable insights for optimizing the volume ratios of iron solutions in leaching processes.

### 4.3 Calculating the reaction order

**Fig. 7:**
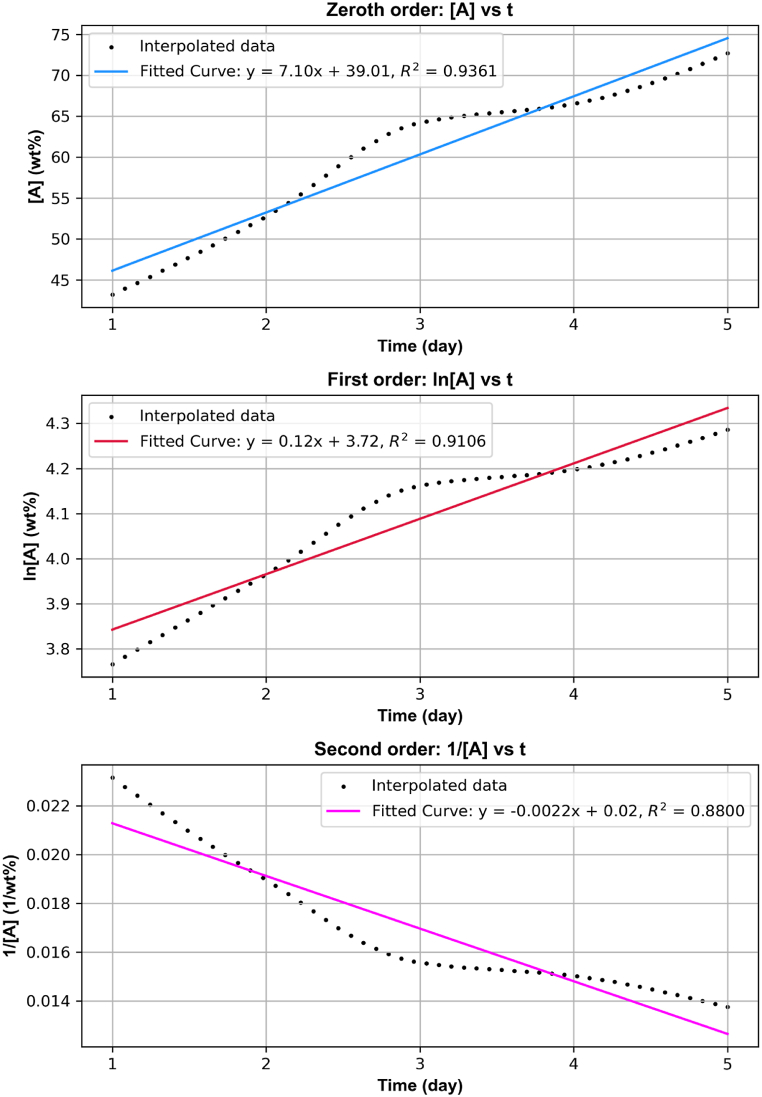
Reaction order for the copper recovery reaction of copper with iron solution.

The series of plots presented in Fig. 7 illustrates the kinetic analysis of the copper extraction reaction using ferrous and ferric solutions. The data of copper extraction with Solution B was taken for this analysis. Each plot provides a unique perspective on how the concentration of copper changes over time under the different assumed reaction orders.

The objective of this analysis was to determine the order of the reaction by examining the linearity and correlation coefficients (R^2^) of different reaction order plots: zeroth, first, and second order. In a zeroth-order reaction, the rate of extraction remains constant over time, independent of the concentration of copper in the solution [57]. In a first-order reaction, the rate of extraction is proportional to the concentration of copper, decreasing as the concentration diminishes over time. In a second-order reaction, the rate of extraction would be proportional to the square of the concentration, leading to a more rapid decrease in rate as the concentration falls.

The first plot, representing the zeroth-order reaction, plots the concentration of copper ([A]) against time (t). The fitted curve, described by the equation y = 7.10x+39.01, shows a linear relationship with a high R^2^ value of 0.9361. This high correlation coefficient indicates that the data points closely follow the fitted line, suggesting that the copper extraction reaction could be zeroth order. Moving to the second plot, which assumes a first-order reaction, the natural logarithm of the copper concentration (ln[A]) is plotted against time. The fitted curve equation y = 0.12x + 3.72 also reveals a linear relationship but with a slightly lower R^2^ value of 0.9106 compared to the zeroth order plot. This indicates that while the first-order assumption provides a reasonably good fit, it is not as strong as the zeroth order. The third plot examines the second-order reaction by plotting the inverse of the copper concentration (1/[A]) against time. The fitted curve, given by y = - 0.0022x + 0.02, shows a linear relationship but with the lowest R^2^ value of 0.8800 among the three plots. This suggests that the second-order assumption is the least accurate fit for the data.

Comparing the R^2^ values across the three plots reveals that the zeroth-order plot, with the highest R^2^ value of 0.9361, provides the best fit. This indicates that the copper extraction reaction with ferrous and ferric solutions is most likely a zeroth-order reaction. Consequently, the rate of copper extraction remains constant over time, independent of the concentration of copper in the solution, providing valuable insights into optimizing the leaching process for maximum efficiency.

Zeroth-order reaction kinetics significantly simplifies the design and operation of batch reactors. When a reaction exhibits zeroth-order kinetics, the rate of product formation remains constant, regardless of the reactant concentrations. This consistency allows engineers to design reactors with predictable outputs and enables straightforward calculations for reactor sizing and operational parameters. Additionally, the predictable behavior of these reactions simplifies the scaling process from laboratory to industrial settings, allowing engineers to anticipate performance in larger systems and ensuring smoother transitions to commercial production. Overall, the application of zeroth-order kinetics leads to greater efficiency, reliability, and safety in various industrial processes.

### 4.4 Optimizing the PCB feed to recover maximum copper

**Fig. 8:**
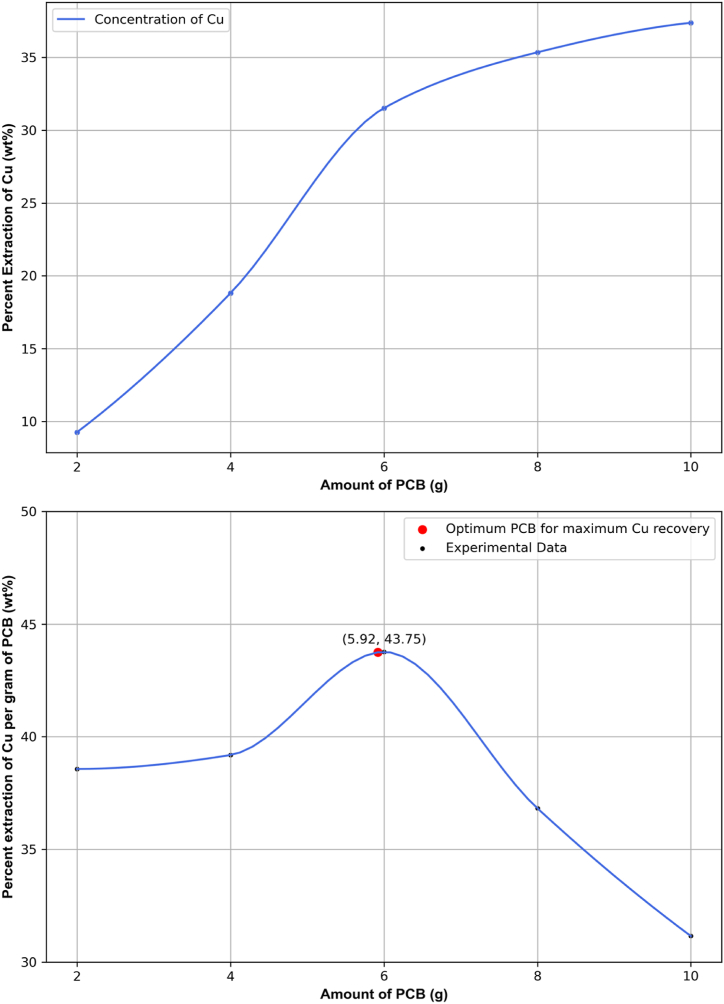
Optimization of PCB feed for maximum copper recovery from experimental data applying SciPy Library of Python Language (additional information is provided in Supplementary Material).

For a given amount of leaching agent, specifically the iron solution used in this study, the total recovery of copper will continue to increase until the reaction ceases when the limiting reactant(s) are depleted. Therefore, there is a maximum quantity of e-waste from which the maximum amount of copper can be extracted using a specific amount of iron solution. This maximization can be achieved computationally. In this study, the SciPy optimization tool of Python programming language was utilized to find the maximum PCB amount for limited leaching agent. This tool is versatile and efficient, supporting a wide range of optimization problems, including linear, nonlinear, constrained, and unconstrained. It provides robust algorithms, from gradient-based solvers to global optimizers, ensuring accuracy and reliability. Seamless integration with libraries like NumPy and Matplotlib enhances data analysis and visualization workflows. Fig. 8 illustrates the total copper recovery and the percent extracted copper per gram of PCB over a 5-day reaction cycle for five different feed samples, ranging from 2 to 10 g of PCB, with an increment of 2 g. Table S4 in the Supplementary data file shows the data for the optimization of PCB amount.

The provided plots illustrate the relationship between the amount of printed circuit board (PCB) e-waste and the percent extraction of copper (Cu) using a fixed amount of leaching agent. In the top plot, the percent extraction of copper is plotted against the amount of PCB e-waste. The graph shows a clear upward trend where the percent extraction of copper increases as the amount of PCB increases from 2 g to 10 g. This indicates that as more e-waste is processed, a higher overall percentage of copper can be extracted. However, this increase tends to taper off slightly as it approaches 10 g, suggesting that the system is nearing its extraction capacity due to the fixed amount of leaching agent. The bottom plot provides a more detailed insight into the efficiency of copper extraction per gram of PCB. Here, the percent extraction of copper per gram of PCB is plotted against the amount of PCB e-waste. Initially, as the amount of PCB increases, the efficiency of copper extraction per gram also increases, reaching a peak at around 5.92 g of PCB, where the percent extraction per gram is approximately 43.75 %. This point is marked as the optimum PCB amount for maximum copper recovery.

Beyond this optimal point, the extraction efficiency per gram of PCB starts to decline. This decrease occurs because the fixed amount of leaching agent becomes insufficient to sustain the maximum extraction rate as the amount of PCB continues to increase. Essentially, the leaching agent becomes the limiting reactant, causing the reaction to slow down and reducing the efficiency of copper extraction per additional gram of PCB. The analysis indicates that there is an optimal amount of PCB e-waste (around 5.92 g) for which the fixed amount of iron solution used in the study can extract the maximum amount of copper most efficiently. Beyond this point, the extraction efficiency decreases because the leaching agent is not sufficient to maintain the high extraction rate. These findings are crucial for optimizing the leaching process, ensuring that the maximum amount of copper can be recovered from e-waste using a specific quantity of leaching agent. This optimization is important for improving the economic and environmental efficiency of the copper extraction process from electronic waste.

## 5 A case study on E-waste management in Dhaka

**Fig. 9:**
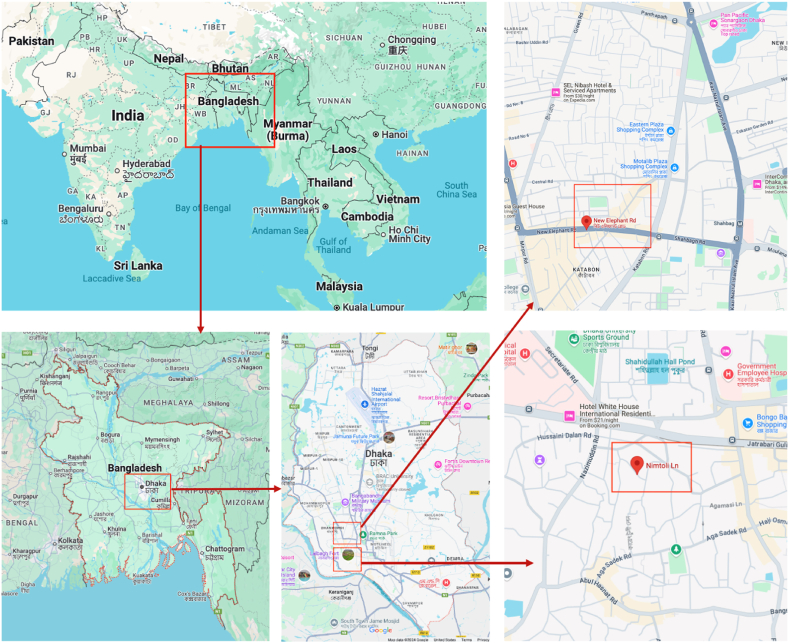
Locations of the survey sites. The images were created from screenshots of Google Map.

Dhaka, a historically well-known hub for industry and technology, generates significant amounts of electronic waste (e-waste), offering valuable insights into urban e-waste management in developing nations. This study examines e-waste accumulation and its economic potential, focusing on two key recycling hubs, Nimtoli and Elephant Road. These locations were selected due to their prominence as major e-waste recycling sites in Dhaka [58]. Fig. 9 shows the location of the survey areas.

Nimtoli, situated in Old Dhaka, hosts approximately 70 shops specializing in the manual dismantling, sorting, and crushing of e-waste. These processes are followed by chemical treatments and metal extraction through burning. These shops operate in cramped spaces within buildings, filled with a variety of discarded electronic items. Shop owners acquire e-waste through auctions from organizations, purchases from hawkers, individuals, retailers, and inter-shop exchanges. On average, each shop procures e-waste valued at 1 to 1.5 million BDT monthly, generating a profit of about 0.1 million BDT. Recycled materials are typically sold to exporters who ship them to China. Although some shop owners are hesitant to disclose trade volumes, most employ 2 to 3 workers who work 8–10 h a day, earning a monthly salary of around 10,000 BDT. Overall, each shop generates sales of approximately 1.12–1.63 million BDT per month. These businesses have been operational for nearly two decades.

Shops on Elephant Road share similarities with those in Nimtoli but differ in their additional focus on repairing and reselling e-waste. These shops primarily handle computer-related components, such as motherboards, monitors, cables, DVD-ROMs, and hard drives. There are two main types of shops in this area: those that exclusively purchase discarded electronics for recycling, similar to Nimtoli, and those that buy functional devices, repair them, and sell them at reduced prices. Typically, these shops employ two workers who work about 10 h a day and earn a monthly wage of 8000 BDT. Like Nimtoli, shop owners here have been in business for nearly 20 years.

### 5.1 Survey findings on E-waste accumulation

The survey data on e-waste accumulation from two different areas of Dhaka indicates that approximately 120 shops in the selected regions collects e-waste ranging from 40 to 2000 kg per month. On average, each shop accumulates about 1025 kg of e-waste monthly in Chankharpool and 520 kg in Elephant Road. Consequently, the total monthly e-waste accumulation is 71,750 kg in Chankharpool and 26,000 kg in Elephant Road, leading to annual accumulations of approximately 861 and 312 tons, respectively. This results in a combined yearly total of around 1173 tons of e-waste in the survey areas. The data also suggests that with an average monthly earning of 1.63 million BDT, the average value of 1545 kg of e-waste translates to a selling price of 1055 BDT per kg for a single shop. However, these figures are likely underestimations, as shop owners tend to underreport the actual data.

The case study findings are presented in Table 3. Table 4 presents the economic aspects of a business model for e-waste recycling in Dhaka which is derived by combining the experimental results and the case study data. The questionnaire (Table S5) and some photographs of the survey sites (Fig. S7) are presented in the Supplementary data file.

**Table 3:** Findings of the case study.

Parameters	Chankharpool	Elephant Road	Total
Average number of shops	70	50	**120**
Range of e-waste accumulation per shop, (kg/month)	50–2000	40–1000	**-**
Average amount of e-waste per shop, (kg/month)	1025	520	**1545**
Total amount of e-waste accumulated, (kg/month)	71,750	26,000	**97,750**
Total amount of e-waste accumulated, (ton/year)	861	312	**1173**
Average earning per shop (million BDT/month)			1.63 million

**Table 4:** Integrating the experimental findings with the insights derived from the survey to plan for a business model.

Parameters	Chankharpool	Elephant Road	Total
Cu amount in e-waste of survey areas, (kg/month)	4305	1560	**5865**
Extractable Cu with iron solution, (kg/month)	1884	683	**2567**
Extractable Cu with iron solution, (ton/year)	23	8	**31**
Estimated earnings from recovered copper, (million BDT/year)	25.91	9.39	**35.30**

### 5.2 Economic potential of copper recovery

In the experiment, it was found that the maximum amount of extractable copper from router PCBs reached 43.77 wt% when using a 50:50 (v/v) iron solution over 5 days. This result highlights the potential for significant copper recovery from electronic waste, particularly when scaled to real-world collection data. According to our field survey, approximately 1173 tons of e-waste are collected annually from the surveyed areas alone. If this e-waste is properly recycled, it could yield around 31 tons of copper. At the current market rate of Tk. 520.28 per pound (or $8.83 per kg), selling the recovered copper could generate more than 35 million BDT annually [59].

**Fig. 10:**
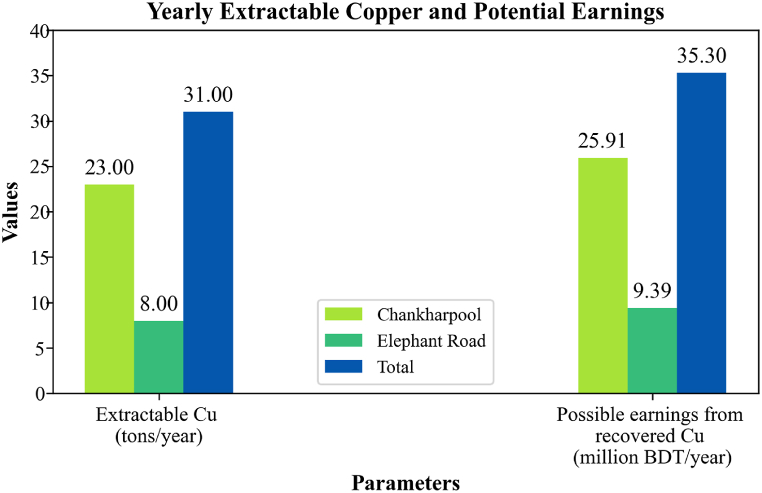
The financial outcomes of the survey.

This figure only reflects the copper recovered from the specific e-waste collected in the surveyed regions, demonstrating the considerable economic potential of e-waste recycling. The projected financial gains from selling the recovered copper, emphasize the broader economic opportunities that could arise from expanding e-waste collection and recycling efforts across larger areas. Thus, this data underscores not only the environmental benefits of recycling but also the potential for substantial revenue generation from metal recovery in e-waste management. Economic outcomes of the survey are presented in Fig. 10.

### 5.3 E-waste management practices and challenges

According to a survey by the Department of Environment of Bangladesh, only 3–5 percent of e-waste is currently collected for processing, with the remaining 95 percent destined for disposal in landfills [60]. Therefore, if 1173 tons of e-waste are being accumulated for recycling annually in the Chankharpool and Elephant Road areas, it can be inferred that approximately 30,000 tons of e-waste are being generated there in total, based on an average recycling rate of 4 percent.

Although shops involved in accumulating and treating e-waste attempt to manage the recycling of hazardous materials, the process is non-scientific and hazardous. During the survey, it was noticed that the workers in both locations were handling e-waste without any personal protective equipment (PPE), posing significant health risks. Moreover, waste materials are stored openly on shelves without any covers or storage boxes, raising serious safety concerns for both the workers and the environment. Table 5 presents the regulatory measures that can be taken for e-waste management and the possible challenges in implementing those measures.

**Table 5:** Specific regulatory measures for e-waste management practices and challenges that might arise in implementing these measures.

Regulatory Measure	Description	Challenges in Implementation
*Extended Producer Responsibility (EPR) Guidelines*	Mandates manufacturers to take responsibility for the collection, recycling, and disposal of their products	Weak enforcement, resistance from manufacturers, and high costs of compliance.
*Hazardous Waste (E-Waste) Management Rules*	Establishes guidelines for e-waste collection, recycling, and disposal under the Bangladesh Environmental Protection Act.	Weak enforcement and compliance, informal sector resistance, and lack of accountability.
*Creation of E-Waste Recycling Infrastructure*	Development of dedicated, modern recycling facilities compliant with environmental standards.	High costs, inadequate infrastructure, and resource constraints for setting up recycling centers.
*Mandatory Use of Personal Protective Equipment (PPE)*	Requires workers to wear protective gear to reduce health risks from hazardous materials.	High cost for small businesses, lack of PPE availability, and non-compliance due to lack of enforcement.
*Improved Public Awareness and Education*	Campaigns to educate workers and the public on the dangers of improper e-waste handling and the importance of recycling.	Lack of widespread awareness and engagement from the public and businesses, insufficient resources for education.

### 5.4 E-waste management initiatives in Bangladesh

E-waste management in Bangladesh is a pressing issue, given the country's rapid technological advancement and increasing electronic consumption. The government of Bangladesh has recognized the severity of this issue and has implemented several measures to improve e-waste management. In recent years, Bangladesh has published the “E-waste Management Rules” to provide a regulatory framework for the handling, recycling, and disposal of electronic waste. These rules are part of the broader efforts under the Bangladesh Environmentally Sustainable Technology (BEST) project, which aims to build dedicated e-waste recycling facilities and enhance the country's overall waste management infrastructure [61].

One of the key initiatives under these rules is the development of Extended Producer Responsibility (EPR) guidelines. On May 19, 2024, Environment, Forest, and Climate Change Minister Saber Hossain Chowdhury announced that the government is working to improve the e-waste management system in Bangladesh [62]. These guidelines will mandate producers to take responsibility for the end-of-life management of their products, encouraging more sustainable manufacturing and recycling practices.

On June 10, 2021, the Department of Environment (DOE) in Bangladesh issued the ‘Hazardous Waste (e-waste) Management Rules, 2021′ under the Bangladesh Environmental Protection Act, 1995, focusing on products outlined in the Schedule and outlining obligations for manufacturers, assemblers, collectors, sellers, and consumers. The regulation took effect immediately upon publication, and it has some key provisions, such as that registered manufacturers and recyclers must obtain environmental clearance and establish collection centers with allocated funds. Certain types of lamps unable to be recycled must be surrendered to designated centers. Collection targets for manufacturers and importers increase annually. Information about traders, sellers, and collection centers must be provided to consumers, and these entities are responsible for accepting and transporting WEEE to designated collection points. Non-compliance with these rules may lead to imprisonment for up to 2 years or a fine of up to 200,000 BDT, or both, in accordance with Section 15(1) of the Bangladesh Environmental Protection Act, 1995. Repeat offenders may face imprisonment ranging from two to ten years or a fine ranging from Taka 200,000 to Taka 1,000,000, or both [63]. Table 6 represents the penalties for non-compliance with the acts.

**Table 6:** Non-compliance penalties of e-waste regulatory acts.

Offense	Penalty	Repeat Offense
**No environmental clearance**	Up to 2 years imprisonment or 200,000 BDT fine	2–10 years of imprisonment or 200,000–1,000,000 BDT fine
**Failure to establish collection centers**	As above	As above

**Fig. 11:**
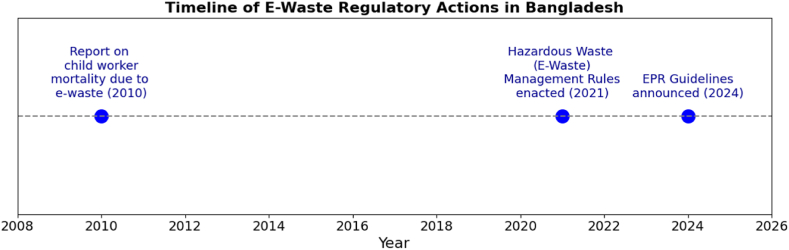
E-waste events and regulatory actions in Bangladesh.

The Hazardous Waste (e-waste) Management Rules 2021 in Bangladesh aim to regulate the handling and disposal of e-waste, ensuring environmental protection and sustainable waste management practices in the country. According to a 2010 report by the Environment and Social Development Organization, more than 15 % of child recycling workers in Bangladesh die during and after the effects of handling e-waste each year, and more than 83 % are exposed to toxic substances [64]. This data highlights not only the severe health risks posed by electronic waste but also the potential burden on the country's healthcare system. From a legal perspective, these findings underscore the urgent need for stricter regulations and enforcement to protect vulnerable populations from hazardous working conditions. Fig. 11 illustrates the regulatory actions related to e-waste events in Bangladesh.

To mitigate the risks associated with e-waste, authorities must take proactive measures. This includes educating workers about the basics of e-waste management and ensuring they use personal protective equipment (PPE) when handling hazardous materials. Additionally, establishing a dedicated oversight committee to enforce these regulations is crucial for maintaining safety standards.

### 5.5 Practical implications of the research findings

The study reveals that by combining field data with lab-based findings, approximately 35 million BDT in annual revenue could be generated from copper recovery in just two small areas of Dhaka. These areas alone accumulate around 31 tons of extractable copper from e-waste per year. The copper content of the e-waste was found to be 6 % in the experiment, of which 43.75 % is extractable with the 50:50 iron solution. According to a study by Roy and his team, Bangladesh produces roughly 3.1 million metric tons (MMT) of e-waste annually, with an estimated annual business potential of 221 million USD, compared to the global figure of 7 billion USD [65]. From these estimates, a viable business model could be developed by implementing systematic recycling and metal recovery from e-waste, offering both economic profit and environmental benefits.

However, such a model must be built on robust government policies and regulations. To increase public awareness, research findings and existing policies should be communicated in a simple, accessible manner, with national television and print media playing a key role [66]. Additionally, the government needs to designate isolated zones for the safe disposal of treated e-waste and promote these disposal sites to educate the public on the dangers of e-waste. Table 7 lists the key findings of the survey in tabulated form.

**Table 7:** Summary of research findings.

Aspect	Data/Insights
Copper Content in E-Waste (Experiment)	6 %
Extractable Copper (50:50 Iron Solution)	43.75 % of total copper content
Extractable Copper (Annual, Survey)	∼31 tons from surveyed areas
Revenue from Copper Recovery	35 million BDT annually from two surveyed areas
E-Waste Accumulation (Survey)	1173 tons annually (combined from Chankharpool and Elephant Road)
Bangladesh E-Waste Production	3.1 million metric tons (MMT) annually
Estimated Business Potential (Bangladesh)	221 million USD annually
Global E-Waste Business Potential	7 billion USD annually
Reaction Kinetics (Zeroth-Order)	- Reaction rate is constant, independent of copper concentration. - Simplifies batch reactor design and scalability. - Enables predictable scaling from lab to industrial setups.
Proposed Actions for E-waste Management	- Develop a business model for recycling e-waste. - Strengthen government policies and regulations. - Raise public awareness. - Designate and promote safe disposal zones.

## 6 Conclusions and future work

The lab experiments highlighted the promise of recovering valuable metals like copper through optimized chemical leaching, offering a greener alternative to traditional methods. A field survey conducted in two key areas of Dhaka also provided crucial insights into local e-waste accumulation, which helped estimate the broader national context.

The study identified a 1:1 mixture of ferrous and ferric sulfate (Solution B) as the most effective leaching agent, achieving a copper extraction efficiency of 72.69 % over five days. Kinetic analysis showed that the process followed zeroth-order kinetics, meaning the extraction rate remained steady as long as the leaching agent and PCB were present, regardless of copper concentration. The optimal conditions for copper recovery involved 5.92 g of PCB in 500 mL of the iron solution, with efficiency declining beyond this threshold. If scaled up within a well-regulated and safety-conscious business model, this method could significantly boost the economy.

The survey findings emphasize the economic potential of copper recovery from Dhaka's 1173 tons of annual e-waste but also highlight the need for robust e-waste management strategies. Within this e-waste, extractable copper is 31 tons whose market value is more than 35 million BDT. Educating recyclers on the dangers of improper handling, encouraging the use of protective gear, and enforcing stricter regulations are essential.

A significant limitation of the study was its narrow focus on copper recovery, overlooking other valuable metals present in PCBs, such as gold, silver, and palladium. Additionally, the study was limited to router PCBs, leaving an opportunity to test the approach on various e-waste types, such as mobile phone or television PCBs, to evaluate the scalability of the process.

Future efforts thus, should aim to expand recovery methods to target multiple valuable metals, improving the economic feasibility of e-waste recycling. Optimizing chemical leaching techniques to enhance extraction efficiencies for a broader range of metals is essential, especially when dealing with diverse types of PCBs. Incorporating tools like Life Cycle Assessment (LCA) and Material Flow Analysis (MFA) in future studies could provide a more holistic understanding of the environmental and economic impacts of recycling since such assessments are scarce in developing nations.

## CRediT authorship contribution statement

**Kaniz Fatema:** Writing – review & editing, Writing – original draft, Visualization, Supervision, Resources, Methodology, Investigation, Formal analysis, Data curation, Conceptualization. **Md Niamul Hassan:** Writing – original draft, Methodology, Investigation, Conceptualization. **Sanjida Hasan:** Writing – original draft, Methodology, Investigation, Formal analysis, Conceptualization. **Hridoy Roy:** Supervision, Methodology, Conceptualization.

## Data availability

Data will be made available on request. For requesting data, please write to the corresponding author.

## Declaration of competing interest

The authors declare that they have no known competing financial interests or personal relationships that could have appeared to influence the work reported in this paper.
