# Supplementary material for: E-waste recycling in an optimized way for copper recovery by leaching and a case study on E-waste generation and management in Dhaka city
Source: Heliyon. 2024 Dec 27;11(1):e41453. doi: 10.1016/j.heliyon.2024.e41453 (PMC11750467; doi:10.1016/j.heliyon.2024.e41453)
Supplement: Multimedia component 1 [file mmc1.docx]

Supplementary Data

# 1. Material Balance for Preparing the Leaching Agent

Ferrous sulfate (FeSO_4_.7H_2_O) and ferric sulfate (Fe_2_(SO_4_)_3_.xH_2_O) were bought from the local market. The molecular weights of these chemicals were mentioned as 278.01 and 399.88 g/mol respectively. To make 100 ml 0.5 M ferrous sulfate solution 14 gm FeSO4.7H_2_O was taken and dissolved in distilled water. Similarly, 20 gm Fe_2_(SO_4_)_3_.xH_2_O was dissolved to make 0.5 M ferric sulfate solution. Table S1 shows the calculation for the required weight of the salts to make the solutions of desired molarity.

Table S1: Calculation of weight of ferrous sulfate and ferric sulfate salt for making 0.5 M solutions.

| **Salt** | **Molecular weight**  **(g/mol)** | **Concentration**  **(M)** | **Volume**  **(ml)** | **Weight to be taken**  **(g)** |
| --- | --- | --- | --- | --- |
| **FeSO_4_.7H_2_O** | 278.01 | 0.5 | 100 | 13.9005 |
| **Fe_2_(SO_4_)_3_.xH_2_O** | 399.88 | 0.5 | 100 | 19.9940 |

These iron sulfate solutions were mixed in different volume ratios for determining the best concentration. For Solution A, the volume ratio was 25 to 75. To prepare this leaching agent, 125 ml of 0.5 M iron (II) sulfate solution and 375 ml of 0.5 M iron (III) sulfate solution were mixed to get a 500 ml iron solution. For Solution B the volumes were 250 ml for both the ferrous sulfate and ferric sulfate solutions. For Solution C, these values were 375 ml and 125 ml respectively. Table S2 shows the volumes of iron (II) and iron (III) solutions to prepare the leaching agents of different concentrations.

Table S2: Calculation of volumes for ferrous sulfate and ferric sulfate solutions for the preparation of Solution A, Solution B, and Solution C.

| **Leaching Agent** | **Ratio of Ferrous Sulfate and Ferric Sulfate**  **(vol%)** | **Volume of Ferrous Sulfate Solution of 0.5 M**  **(ml)** | **Volume of Ferric Sulfate Solution of 0.5 M**  **(ml)** | **Total Volume of Iron Solution**  **(ml)** |
| --- | --- | --- | --- | --- |
| **Solution A** | 25:75 | 125 | 375 | 500 |
| **Solution B** | 50:50 | 250 | 250 | 500 |
| **Solution C** | 75:25 | 375 | 125 | 500 |

# 2. Experimental work

## 2.1. Collection of Samples

Router PCB e-waste, sourced from a local e-scrap store in Chankharpool, Old Dhaka, was transported to the laboratory without any prior mechanical or physical separation. The price was 50 Taka per PCB. Upon arrival, non-metallic components were manually removed as thoroughly as possible. The PCBs were cleaned, rinsed, and then dried in an oven at 140°C for 48 hours. After drying, the PCBs were finely ground using an electronic blender to produce a homogeneous material, which was subsequently stored in an airtight zipper bag.  Figure S1 shows the process block diagram for the pretreatment of PCB.


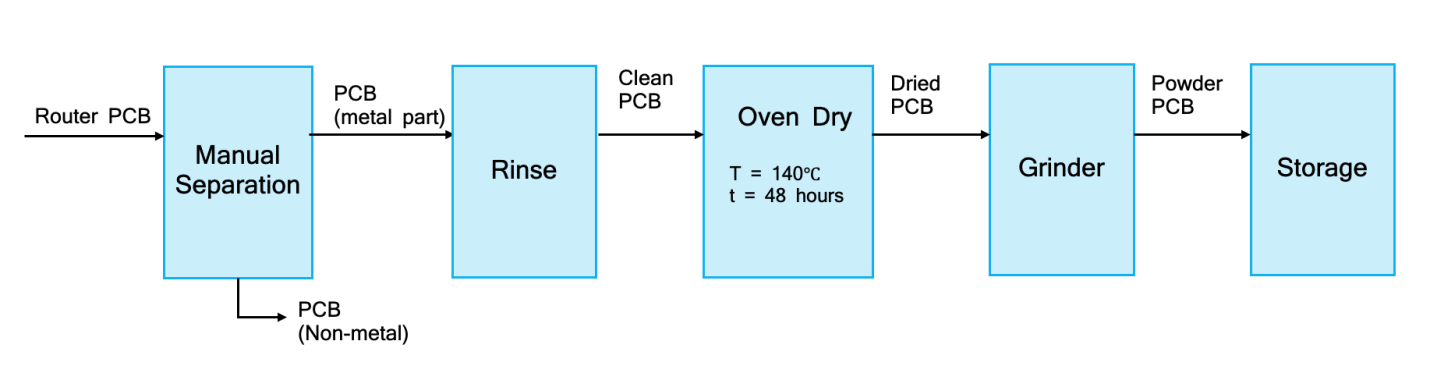


Figure S1: The process of pretreatment of the PCB.

## 2.2. Determination of Copper Content of Router PCB Sample

Top of FormBottom of FormIn a fume hood, 30 mL of 37.25 wt.% HCl was added to a beaker, followed by the gradual addition of 10 mL of 69 wt% HNO₃. The initially clear solution was left to stand for 10 minutes, during which it developed an orange-red hue. Subsequently, 1 g of router PCB powder was dissolved in 40 mL of the freshly prepared aqua regia and heated at 180°C for 20 minutes. The PCB mass was the basis for the experiment and the volume of aqua regia was sufficient to dissolve the e-waste. After heating, the solution was diluted to 120 mL by adding 80 mL of distilled water. The dilution was necessary for the concentration analysis step. The concentration of recovered copper was then measured using Atomic Absorption Spectrophotometry (AAS). Aqua regia is a strong leaching agent that is capable of extracting any metal due to its swift leaching kinetics [1].Figure S2 illustrates the determination of the copper content of the PCB sample collected from old Dhaka.


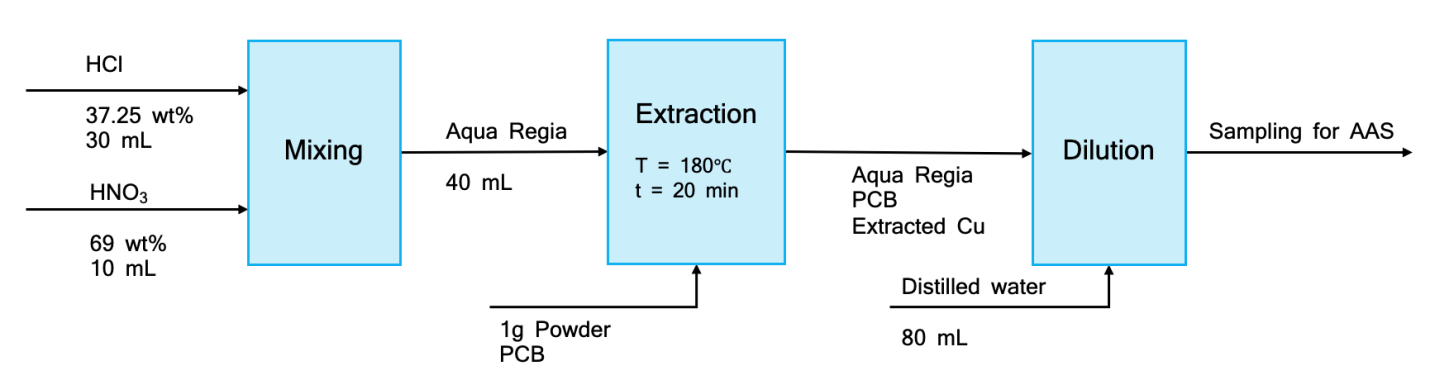


Figure S2: Procedure for the determination of copper content in the PCB sample.

Using aqua regia to extract metals from e-waste presents several significant challenges. It is highly corrosive and produces toxic fumes, posing serious health and safety risks [2]. The process generates hazardous waste, which is costly and difficult to dispose of responsibly, negatively impacting the environment. NO_X_ generated from the aqua regia process can potentially pollute the environment and requires repeated evaporation steps to destroy nitro compounds[3]. Additionally, due to the rapid reaction of its components, aqua regia decomposes quickly, losing its effectiveness, which means it must be freshly prepared for each use. These operational challenges, including stringent safety protocols, handling risks, and the instability of aqua regia, make its use complex and costly, highlighting the need for careful management and consideration of alternative methods.

## 2.3. Establishing Optimum Time for Extraction of Copper with Iron Solution

To develop an alternative reagent for copper extraction, a solution comprising ferrous sulfate and ferric sulfate was prepared, considering the relatively lower environmental impact of iron-based solutions. Although this leaching agent is less hazardous compared to aqua regia, careful handling is still required, as both iron (II) and iron (III) solutions can irritate the respiratory tract, eyes, and skin through inhalation or direct contact. In severe or prolonged cases, ferrous sulfate exposure can damage blood vessels. Additionally, the solution must be maintained at a stable temperature around 25ºC, as temperature fluctuations can lead to product oxidation, generating sulfur oxides that contribute to greenhouse gas emissions[4].Due to its acidic nature, any spillage of ferric sulfate solution may cause localized damage to vegetation. It should not be discharged directly into rivers or drains, as dilution to concentrations below approximately 1% results in the formation of ferric hydroxide. In contact with metals, this compound produces hydrogen gas, which, when combined with air, can create explosive mixtures.

Firstly, 14 grams of FeSO_4_.7H_2_O, with a molecular weight of 278.01, was dissolved in 100 mL of water to create a 0.5 M Ferrous solution. The weight of salt was sufficient to get a homogeneous solution. Similarly, 20 grams of Fe_2_(SO_4_)_3_.xH_2_O, with a molecular weight of 399.88, was dissolved in 100 mL of water to prepare another solution containing ferric ions of 0.5 M. These solutions were then combined in a 1000 mL beaker to form the final leaching agent. The iron salts were bought from the local market and their molecular masses along with the formulae were mentioned by the manufacturers. Table S1 in the Supplementary Data file shows the material balance for preparing the leaching agent.

Subsequently, 200 mL of this iron solution was used, into which 10 grams of crushed PCB powder was introduced. An excess amount of PCB was used to ensure the saturation of the solution with recovered copper within a specific timeframe. The mixture was heated to 32°C and stirred using a magnetic stirrer at 180 rpm. To determine the optimal duration for copper leaching, the extraction process was extended to a total of 10 days. Samples were taken from the solution on the 5th day and the 10th day for analysis to quantify the extracted copper content. Figure S3 shows the block diagram of the extraction process by which the optimum duration of the reaction was determined. The compositionof the leaching agent was optimized in subsequent steps by observing the outcomes, which was a trial-and-error procedure.


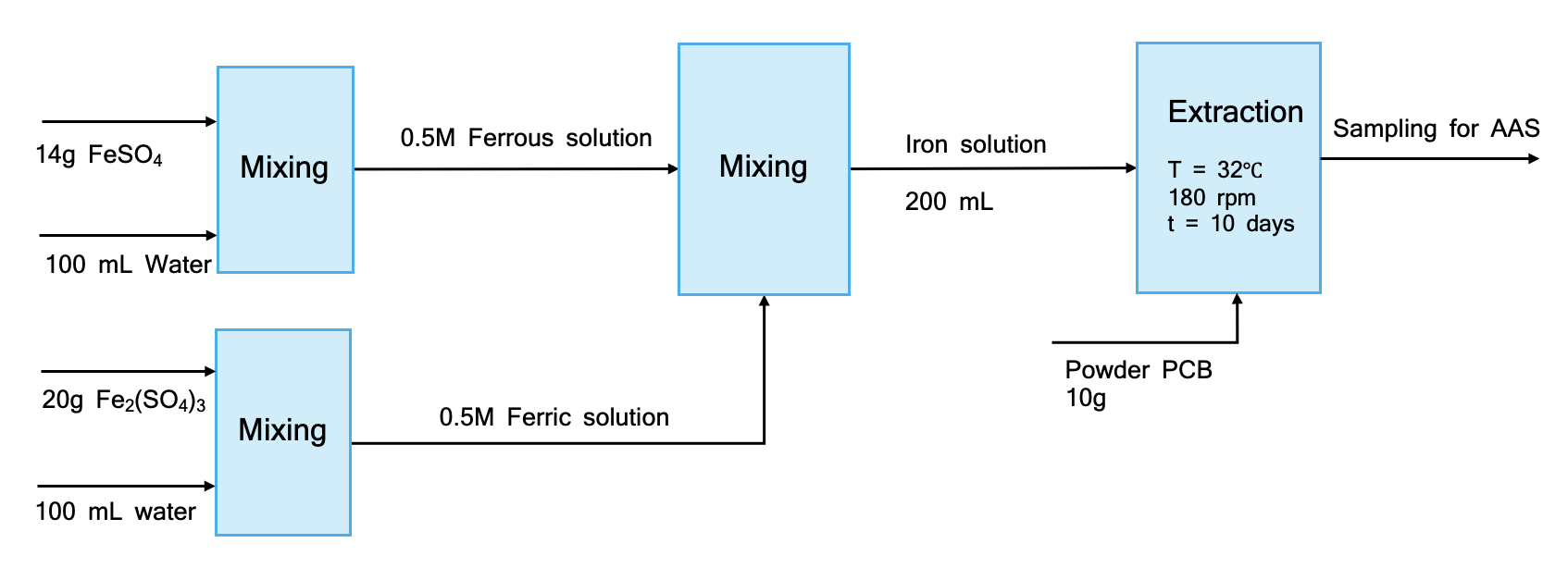


Figure S3: Procedure of determining the optimum time for extraction.

## 2.4. Optimizing Copper Extraction - Effects of Iron Solution Composition

Three sets of iron solutions with varying ratios of ferrous sulfate to ferric sulfate were prepared: Solution A (25% ferrous sulfate, 75% ferric sulfate), Solution B (50% ferrous sulfate, 50% ferric sulfate), and Solution C (75% ferrous sulfate, 25% ferric sulfate). All the percentages are in volume here. The ratios of the iron-based leaching agents were adjusted by increasing the ferrous sulfate content from one-quarter to three-quarters to observe how changes in the proportions of iron (II) and iron (III) solutions affected copper extraction.Each solution, at a concentration of 0.5 M and a volume of 500 mL, was mixed with 2 grams of crushed PCB and agitated on a shaker at 120 rpm for 5 days at room temperature. An excess amount of solution was used to ensure that each leaching agent could extract the maximum possible amount of copper from the PCB. The leaching was limited to a five-day period because extending it to 10 days showed minimal improvement in copper recovery in procedure C. This approach allowed for an effective comparison of the different solutions' efficiencies. Samples were taken every 24 hours and analyzed for copper recovery using AAS. Figure S4 shows the method of extracting copper with Solution A. For Solution B and Solution C, the volume ratios of the ferrous solution and the ferric solution were 50:50 and 75:25, respectively. The data for this step is presented in Table S2 in the Supplementary data file.


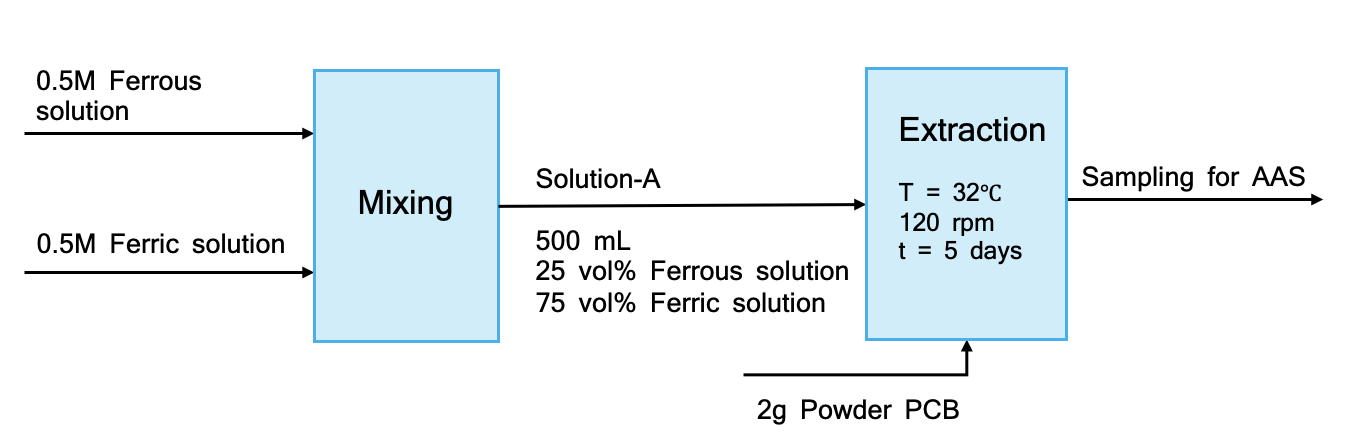


Figure S4: Procedure of determining the extracted copper with Solution A. Solution B and Solution C were prepared in the same way by changing the volume ratio of ferrous sulfate and ferric sulfate solutions.

## 2.5. Optimization of PCB Quantity

To prevent the loss of e-waste as a feed for the leaching process, five identical optimum iron solutions were prepared, each with a concentration of 0.5 M and a volume of 500 mL. Crushed PCB was then added to these solutions in varying amounts of 2, 4, 6, 8, and 10 grams, respectively. The mixtures were thoroughly mixed and placed on a shaker rotating at 120 rpm at room temperature. After 5 days, samples were taken from each solution and analyzed using AAS. The concentration of copper in these five different leaching environments was measured, revealing the maximum amount of copper extracted per gram of PCB. Figure S5 illustrates the method of finding the optimum amount of PCB for maximum recovery of copper.


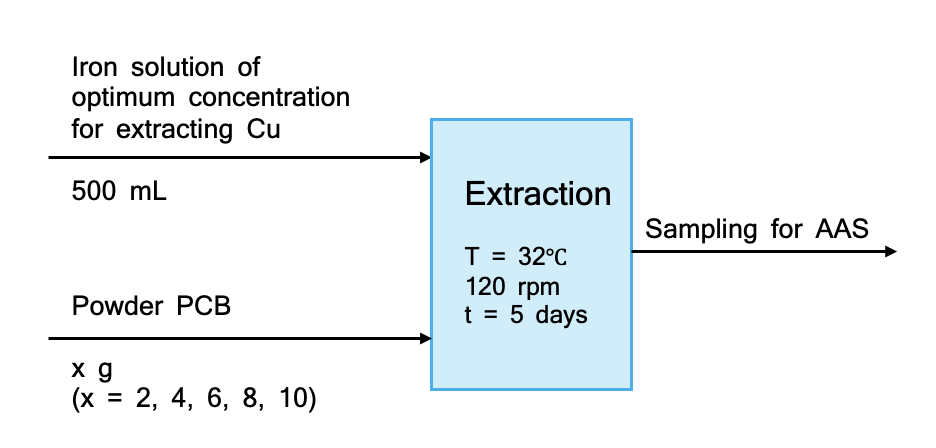


Figure S5: Procedure of finding the optimum amount of PCB for maximum copper extraction.

Figure S6 illustrates the raw materials and the extraction process of copper in iron solutions. The store-bought PCB and their powder form after physical treatment, extraction of copper with aqua regia and iron solution, and the iron solutions of different compositions for leaching can be seen in this Figure. It is to be noted that all the quantities used in the methodology were determined through a combination of literature review and preliminary experiments, though only the final concentrations and outcomes are reported here. The molarity of the iron (II) and (III) solutions was based on the solubility limits of commercially available iron salts.

| 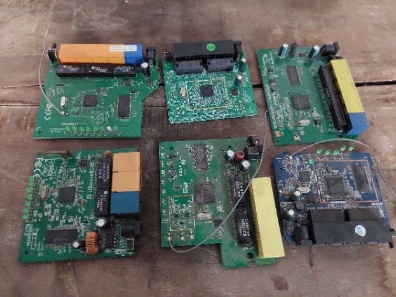  (a) | | 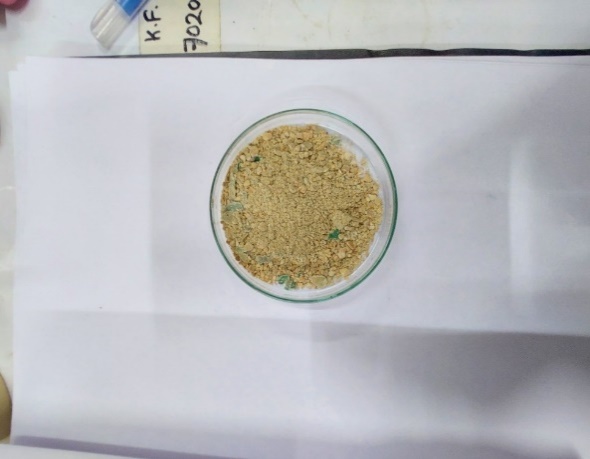  (b) | 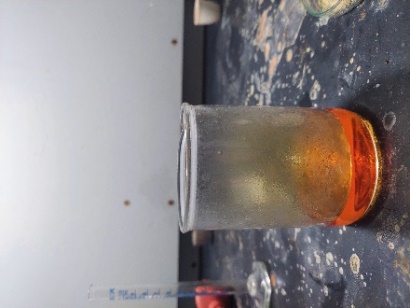  (c) |
| --- | --- | --- | --- |
| 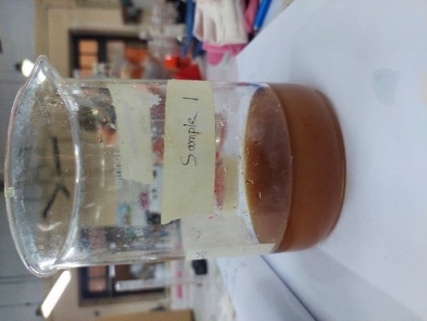  (d) | 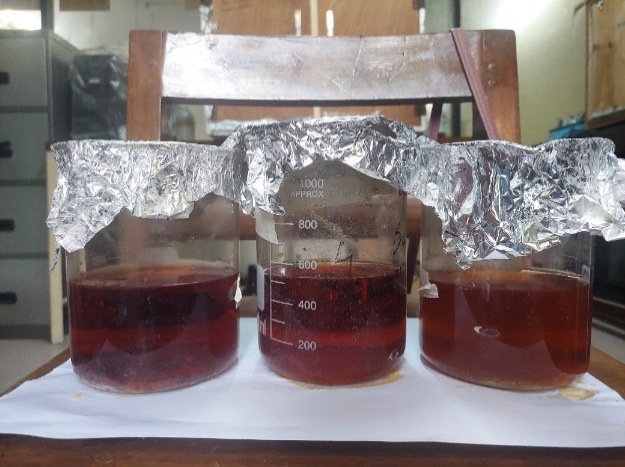  (e) | | |

Figure S6: Copper extraction from e-waste (a) Store-bought PCBs (b) PCBs after physical treatment (c) Leaching with aqua regia to know the copper content (d) Leaching with iron solution to find the optimum duration for the reaction(e) PCB in iron solutions of different compositions to know the maximum copper recovery.

# 3. Data for Optimized Extraction

Table S3: Extracted copper inSolution A, Solution B, and Solution C.

| **Day** | **Percent Extraction of Cu by Solution A,**  **wt%** | **Percent Extraction of Cu by Solution B,**  **wt%** | **Percent Extraction of Cu by Solution C,**  **wt%** |
| --- | --- | --- | --- |
| **1** | 58.52 | 43.22 | 29.85 |
| **2** | 62.64 | 52.74 | 31.91 |
| **3** | 63.81 | 64.21 | 36.99 |
| **4** | 65.36 | 66.52 | 39.18 |
| **5** | 67.26 | 72.69 | 44.93 |

Table S4: Optimization of PCB for the maximum recovery of copper.

| **PCB weight, g** | **Percent Extraction of Cu by 50:50 Iron Solution,**  **wt%** | **Percent Extraction of Cu by 50:50 Iron Solution,**  **wt%/gm of PCB** |
| --- | --- | --- |
| **2.0** | 9.257 | 38.57 |
| **4.0** | 18.809 | 39.19 |
| **6.0** | 31.515 | 43.77 |
| **8.0** | 35.347 | 36.82 |
| **10.0** | 37.377 | 31.15 |

# 4. Link for Python Codes

<https://github.com/kfatema10/E-wasteRecycle>

# 5. SciPy Package

SciPy is an open-source Python library for scientific and technical computing, built on top of NumPy, the fundamental package for scientific computing in Python[5], [6]. Developed in 2001 by Travis Oliphant, SciPy extends NumPy's capabilities, offering advanced functions for optimization, integration, interpolation, linear algebra, signal processing, and more, making it indispensable in data science, machine learning, and scientific research.

SciPy's powerful features include tools for robust optimization algorithms (minimizing or maximizing functions), solving systems of equations, matrix decompositions, and handling large, sparse matrices efficiently[7]. It is also widely used in statistical analysis, Fourier transforms, and solving ordinary differential equations (ODEs), which are crucial for modeling dynamic systems.SciPy is a preferred choice in scientific computing due to its versatility, efficiency, and seamless integration with other Python libraries like Pandas, Matplotlib, and scikit-learn. Its large user community, extensive documentation, and active development ensure strong support and resources for engineers, researchers, and data scientists solving complex computational problems.

In this study, the optimization was performed using the default BFGS (Broyden–Fletcher–Goldfarb–Shanno) method, a gradient-based algorithm. BFGS is part of the quasi-Newton family of methods, which estimate the Hessian matrix (the second derivatives of the function) through gradient calculations. This approach makes BFGS particularly effective for optimizing functions that are smooth and differentiable.

# 6. Survey Questionnaire

Table S5 lists the questions for the survey.

Table S5: Survey questionnaire

| 1. Information of Respondent | |
| --- | --- |
| i) Name:  ii) Gender:  iii) Age:  iv) Education:  v) Number of years working in this profession:  vi) Working area:  vii) Working Hours per day:  viii) Monthly Income:  ix) Family size:  x) Other (if relevant): | |
| 2. Information on E-waste Collection | |
| i) Please name the used electronic items that you collect, segregate, salvage or recycle. |  |
| ii) State the number of units or weight of each type of e-waste that you collect and/or sell to dismantlers per day or per month. |  |
| iii) Do you use any safety measures while collecting, segregating, dismantling the waste? If you do, please mention. |  |
| iv) Do you know about the harmful impact of e-waste on human health and the environment? |  |
| v) Do you know anything about recycling e-waste? |  |
| vi) Do you know anything about safe disposal of e-waste? |  |
| vii) Do you follow environmental regulations imposed by the government? |  |
| viii) Did you meet with any accident while handling e-waste? |  |

Figure S7 highlights various e-waste collection shops in Dhaka where the survey was conducted.


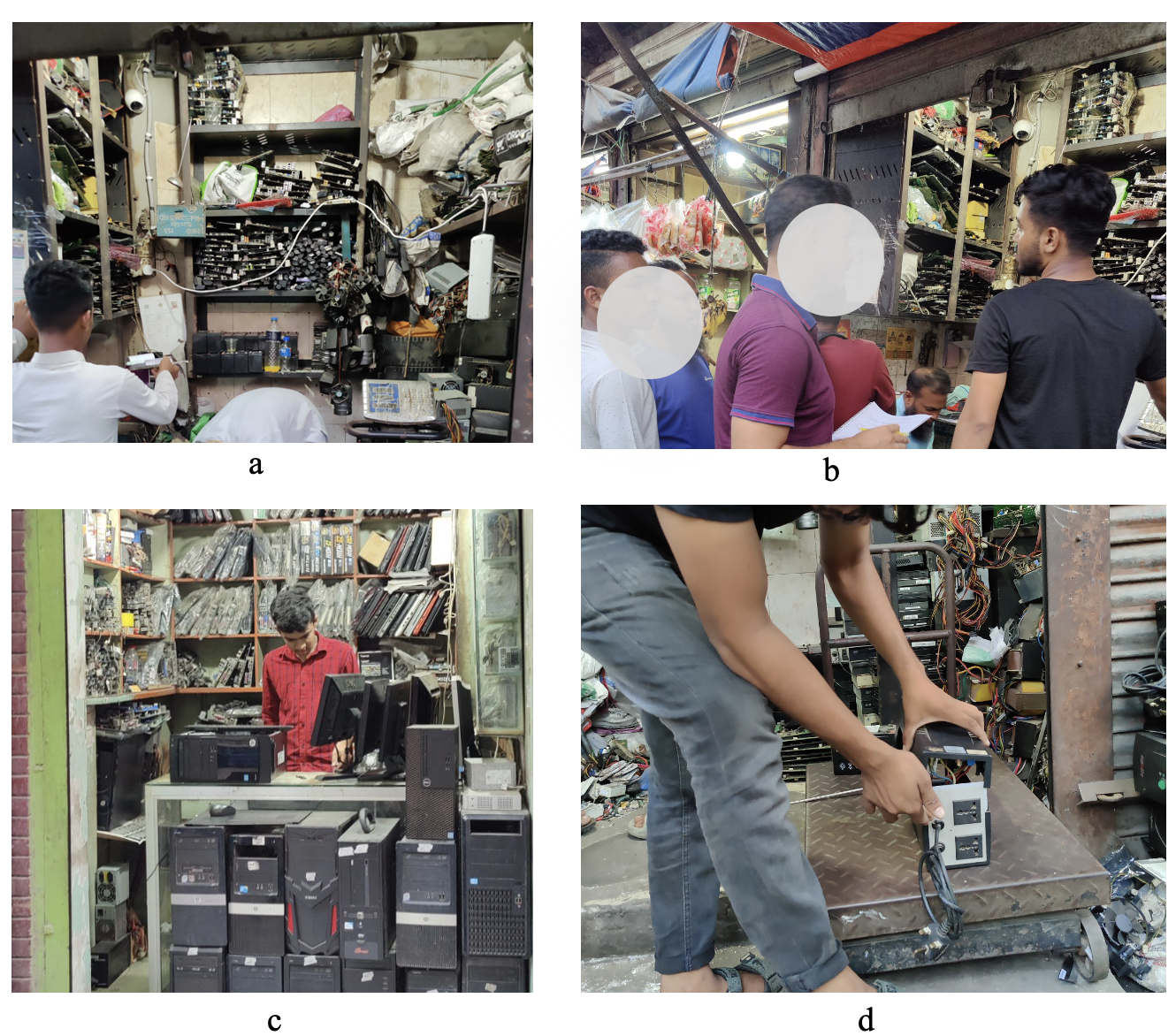


Figure S7: (a), (b), (c)A fewe-waste collection shopsin Dhaka where old electronic units are stacked openly on shelves and floors (d) Inspecting old UPS units without taking appropriate safety precautions prior topurchasing from customers.

# References

[1] M. Mehedi Hasan Rocky, I.M.M. Rahman, M. Endo, H. Hasegawa, Comprehensive insights into aqua Regia-Based hybrid methods for efficient recovery of precious metals from secondary raw materials, Chem. Eng. J. (2024) 153537. https://doi.org/10.1016/j.cej.2024.153537.

[2] P.R. Yaashikaa, B. Priyanka, P. Senthil Kumar, S. Karishma, S. Jeevanantham, S. Indraganti, A review on recent advancements in recovery of valuable and toxic metals from e-waste using bioleaching approach, Chemosphere 287 (2022) 132230. https://doi.org/10.1016/j.chemosphere.2021.132230.

[3] Y. Chen, Q. Qiao, J. Cao, H. Li, Z. Bian, Precious metal recovery, Joule 5 (2021) 3097–3115. https://doi.org/10.1016/j.joule.2021.11.002.

[4] FERROUS SULFATE, (n.d.). https://us.vwr.com/assetsvc/asset/en_US/id/8268740/contents (accessed September 8, 2024).

[5] T.E. Oliphant, Guide to NumPy, Continuum Press, 2015.

[6] P. Virtanen, R. Gommers, T.E. Oliphant, M. Haberland, T. Reddy, D. Cournapeau, E. Burovski, P. Peterson, W. Weckesser, J. Bright, S.J. van der Walt, M. Brett, J. Wilson, K.J. Millman, N. Mayorov, A.R.J. Nelson, E. Jones, R. Kern, E. Larson, C.J. Carey, İ. Polat, Y. Feng, E.W. Moore, J. VanderPlas, D. Laxalde, J. Perktold, R. Cimrman, I. Henriksen, E.A. Quintero, C.R. Harris, A.M. Archibald, A.H. Ribeiro, F. Pedregosa, P. van Mulbregt, SciPy 1.0: fundamental algorithms for scientific computing in Python, Nat. Methods 17 (2020) 261–272. https://doi.org/10.1038/s41592-019-0686-2.

[7] SciPy User Guide — SciPy v1.14.1 Manual, (n.d.). https://docs.scipy.org/doc/scipy/tutorial/index.html (accessed September 8, 2024).
